# Mentalization-based treatment for adolescents with conduct disorder (MBT-CD): protocol of a feasibility and pilot study

**DOI:** 10.1186/s40814-021-00876-2

**Published:** 2021-07-02

**Authors:** Svenja Taubner, Sophie Hauschild, Lea Kasper, Michael Kaess, Esther Sobanski, Thorsten-Christian Gablonski, Paul Schröder-Pfeifer, Jana Volkert

**Affiliations:** 1 Institute for Psychosocial Prevention, University Hospital Heidelberg, University of Heidelberg, Bergheimer Str. 56, D-69115 Heidelberg, Germany; 2 Psychological Institute, University of Heidelberg, Heidelberg, Germany; 3 Clinic of Child and Adolescent Psychiatry, University Hospital Heidelberg, Heidelberg, Germany; 4 University Hospital of Child and Adolescent Psychiatry and Psychotherapy, University of Bern, Bern, Switzerland; 5 Department of Pediatric and Adolescent Psychiatry and Psychotherapy, University Medical Center Johannes Gutenberg University Mainz, Mainz, Germany; 6 University Klagenfurt, Klagenfurt, Austria

**Keywords:** Mentalization-based treatment, Mentalizing, Conduct disorder, Oppositional defiant disorder, Adolescents, Feasibility, Antisocial behavior

## Abstract

**Background:**

Conduct disorder (CD) is a complex mental disorder characterized by severe rule-breaking and aggressive behavior. While studies have shown that several therapeutic interventions are effective in treating CD symptoms, researchers call for treatments based on etiological knowledge and potential patho-mechanisms. Mentalization-based treatment (MBT) may represent such a treatment approach: Studies have shown that individuals with CD show mentalizing deficits and that mentalizing might represent a protective factor against the development of the disorder. As MBT focuses on the understanding of social behavior in terms of mental states, fostering mentalizing might help CD individuals to (re)gain an adaptive way of coping with negative emotions especially in social interactions and thus reduce aggressive behavior. For this purpose, MBT was adapted for adolescents with CD (MBT-CD). This is a protocol of a feasibility and pilot study to inform the planning of a prospective RCT. The primary aim is to estimate the feasibility of an RCT based on the acceptability of the intervention and the scientific assessments by CD individuals and their families indicated by quantitative and qualitative data, as well as based on necessary organizational resources to conduct an RCT. The secondary aim is to investigate the course of symptom severity and mentalizing skills.

**Methods:**

The bi-center study is carried out in two outpatient settings associated with university hospitals (Heidelberg and Mainz) in Germany. Adolescents aged between 11 and 18 years with a CD or oppositional defiant disorder (ODD) diagnosis are included. Participants receive MBT-CD for 6 to 12 months. The primary outcome of the feasibility study (e.g., recruitment and adherence rates) will be descriptively analyzed. Multilevel modeling will be used to investigate secondary outcome data.

**Discussion:**

Fostering the capacity to mentalize social interactions triggering non-mentalized, aggressive behavior might help CD individuals to behave more adaptively. The feasibility trial is essential for gathering information on how to properly conduct MBT-CD including appropriate scientific assessments in this patient group, in order to subsequently investigate the effectiveness of MBT-CD in an RCT.

**Trial registration:**

ClinicalTrials.gov, NCT02988453. November 30, 2016

**Sources of monetary support:**

Dietmar Hopp Stiftung, Heidehof Stiftung

**Recruitment status:**

Recruitment complete and intervention complete, follow-up assessments ongoing (Heidelberg). Recruitment and assessments ongoing (Mainz).

**Primary sponsor, principal investigator, and lead investigator in Heidelberg:**

Svenja Taubner is responsible for the design and conduct of MBT-CD intervention and feasibility and pilot study, preparation of protocol and revisions, and publication of study results.

**Secondary sponsor and lead investigator in Mainz:**

Esther Sobanski is responsible for the recruitment and data collection in the collaborating center Mainz

**Recruitment country:**

Germany

**Health condition studied:**

Conduct disorder, oppositional defiant disorder

**Intervention:**

Mentalization-based treatment for conduct disorder (MBT-CD): MBT-CD is an adaptation of MBT for Borderline Personality Disorder. This manualized psychodynamic psychotherapy focuses on increasing mentalizing, i.e., the ability to understand behavior in terms of mental states, in patients. MBT-CD includes weekly individual sessions with the patient and monthly family sessions.

**Key inclusion and exclusion criteria:**

Included are adolescent individuals with a diagnosis of conduct disorder or oppositional defiant disorder aged between 11 and 18 years.

**Study type:**

Feasibility and pilot study (single-group)

**Date of first enrollment:**

19.01.2017

**Study status:**

The trial is currently in the follow-up assessment phase in Heidelberg and in the recruitment and treatment phase in Mainz.

**Primary outcomes:**

Acceptability of MBT-CD intervention (as indicated by recruitment rates, completion rates, drop-out rates, treatment duration, oral evaluation), acceptability of scientific assessments (as indicated by adherence, missing data, oral evaluation), and necessary organizational resources (scientific personnel, recruitment networks, MBT-CD training and supervision) to estimate feasibility of an RCT

**Secondary outcomes:**

Adolescents’ symptom severity and mentalizing ability

**Protocol version:**

20.08.2020, version 1.0

**Supplementary Information:**

The online version contains supplementary material available at 10.1186/s40814-021-00876-2.

## Background and rationale

Conduct disorder (CD) is a severe and complex mental disorder most common in adolescence. It is defined as a “repetitive and persistent pattern of behavior in which the basic rights of others or major age-appropriate societal norms or rules are violated” [1]. Epidemiological studies have shown that about 5-10% of all children and adolescents meet the criteria for CD, while boys are more likely to be diagnosed than girls [2]. CD is a serious risk factor for the development of antisocial personality disorder (ASPD). More than 50% of men with ASPD fulfilled criteria for CD prior to the age of 15 [3]. Moreover, CD is often comorbid with attention deficit hyperactivity disorder [4] and is associated with an increased risk for the development of a number of other mental disorders, including anxiety disorders, depression, substance use disorder, and bipolar disorder [5]. The following environmental factors have been identified to be associated with an increased risk for the development of CD [6]: dysfunctional parent-child interactions, critical life events such as parental divorce, parental loss, as well as early neglect, physical and sexual abuse [6–8]. The accumulation of risk factors further increases the risk for the development of CD [9]. At the same time, protective factors like intelligence or social support can help reduce the risk for dysfunctional development [10]. Based on these findings, there is consensus that the emergence of CD is complex [11], yet still, very little is known about the mechanisms contributing to the development and maintenance of CD subsequent to the exposure to the identified risk factors.

So far, meta-analyses have shown that a number of interventions are effective in reducing CD symptoms [12, 13]. Among these, cognitive-behavioral therapy, social skills training, parent training, and multi-systemic therapy are regarded as evidence-based treatments [12, 14]. They differ in their involvement of parents or peers, but their focus on CD symptom management is common to all. However, effect sizes are small, conduct problems oftentimes persist and it remains unclear which treatment works best for whom and why [12]. Moreover, drop-out rates of around 20% [12] and low motivation to seek treatment in the first place render the effective and sustainable treatment of CD pathology difficult. Importantly, interventions are so far lacking a comprehensive etiological understanding of CD, which may entail the failing of targeting relevant mechanisms contributing to the development and maintaining of CD symptoms. In line with this notion, authors call for specification of interventions to target specific individual or subgroup deficits [11, 12] and base them on knowledge about underlying patho-mechanisms [11].

Recently, it has been shown that CD alongside many other mental disorders is related to dysfunctions in mentalizing [15]. Mentalizing describes an individual’s imaginative ability to perceive one’s own and other’s behavior as the product of affective and cognitive mental states [16]. Taubner and colleagues [17] showed that adolescents with CD have a significantly lower mentalizing capacity compared to adolescents with no CD. These findings were replicated in a later study by Cropp et al. [18]. Moreover, focusing more on a developmental perspective fostering insight into possible patho-mechanisms, studies showed that mentalizing mediates the relationship between childhood maltreatment and externalizing problems: Taubner and colleagues [19, 20] found that mentalizing (partially) mediated the relationship between childhood maltreatment and potential for violent behavior in adolescence (14-21 years, [19]; 15-18 years, [20]). Similarly, Ensink and colleagues [21] found that mentalizing partially mediated the link between childhood sexual abuse and externalizing problems, such as rule-breaking and aggressive behavior, in children aged between 7 and 12. Moreover, investigating adolescent PTSD patients, Abate and colleagues [22] found that hyper mentalizing mediated the link between trauma and aggression in female PTSD inpatients. Taken together, results indicate that mentalizing may serve as a protective factor against externalizing behaviors while dysfunctional mentalizing may not only be part of CD pathology but also etiology.

The relation between limited (inhibited or biased) mentalizing and aggressive behavior in CD may be explained by different phenomena: Firstly, if mentalizing is inhibited, the “violence inhibition mechanism” as described by Blair [23] might be impaired: According to the author, violent behavior is normally inhibited when we see and empathize with others’ distress. If however mentalizing is inhibited, and consequently, individuals have difficulties to recognize others’ distress, the threshold for aggression and violent behavior might be lowered. Secondly, if mentalizing is negatively biased, aggressive behavior may be elicited due to a more hostile “social information processing” [24–26] in these adolescents, characterized by a tendency to attribute hostile intent upon neutral or even positive social signals. As we can train and change the capacity to mentalize, a treatment focusing on enhancing mentalizing might thus be able to target a relevant patho-mechanism of CD.

In sum, we assume that a psychological intervention with a focus on improving mentalizing in the adolescent and his/her family can improve CD pathology as effective or even more sustainably than interventions focusing on symptom management only.

For this purpose, the authors have developed mentalization-based treatment (MBT) for CD (MBT-CD). MBT-CD is a further development of MBT, which is evidence-based for patients with borderline personality disorder. MBT has already been adjusted successfully to working with adolescents with self-harm [27]. MBT-CD focuses on the development of a basic understanding of interpersonal situations and emotions, and specific mentalizing deficits that trigger antisocial and aggressive behavior. In order to make this new treatment available for the adolescent population, we need to determine its effectiveness by conducting a randomized controlled trial (RCT). In line with the SPIRIT ([28], for an overview of included items please see Additional file 1) and Consolidated Standard of Reporting Trials (CONSORT) statement [29], this protocol of a feasibility study is aimed at enhancing transparency and quality in gathering information to develop and appropriately conduct the protocol of a future RCT.

## Methods/design

### Aims

The primary aim of the feasibility and pilot study is to estimate the feasibility of an RCT on MBT-CD based on acceptability of the intervention (indicated by recruitment rates into treatment, drop-out rates and completer rates, and adolescents’ oral evaluation), acceptability of the scientific assessments by the patients and family (indicated by adherence rates to the scientific assessments, and adolescents’ oral evaluation), and organizational resources needed to conduct an RCT in an outpatient only and a combined outpatient and inpatient setting in the future. The secondary aim is the examination of CD-symptom severity over time, the course of aggressive and antisocial behavior, and changes in mentalizing. The primary aim of the future RCT will be to determine the effectiveness of MBT-CD in terms of CD symptom severity, levels of aggression, and antisocial behavior as well as an appropriate comparison group. The secondary aim of the RCT will be to investigate the change in a proposed patho-mechanism (i.e., mentalizing) and general symptom severity through MBT-CD.

### Design

**Fig. 1:**
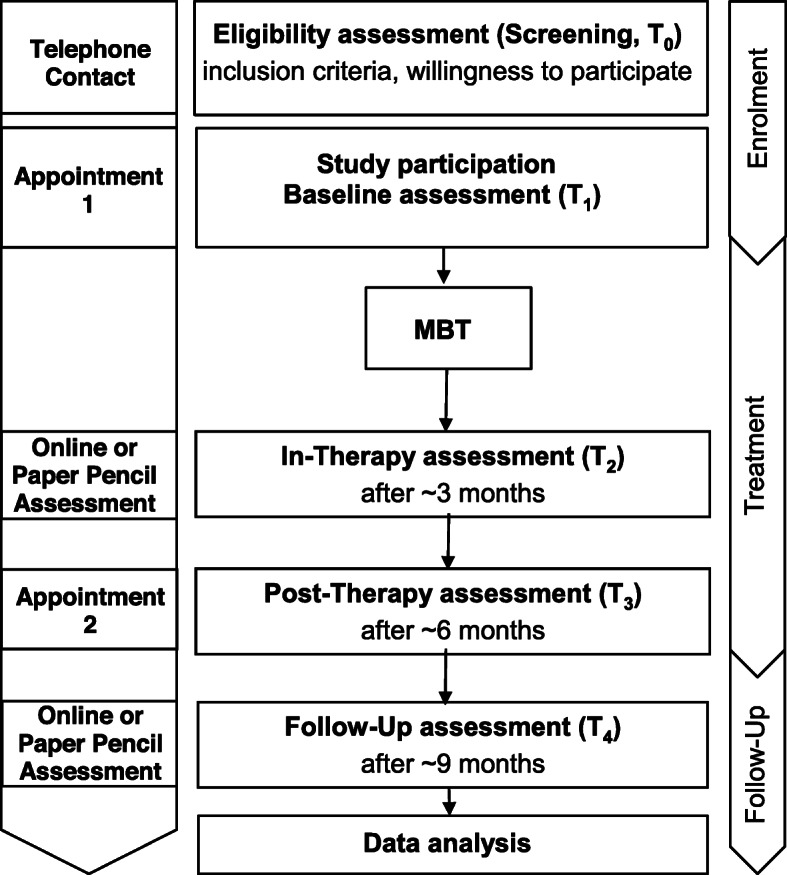
Study flow chart. Sequence of steps from eligibility assessment to data analysis in both settings

The study is a feasibility and pilot trial to form the basis for a future, prospective RCT. The study is carried out at two treatment centers in Heidelberg (Institute for Psychosocial Prevention) and Mainz (Pediatric and Adolescent Psychiatry Mainz), Germany. Adolescent participants receive MBT-CD over the course of 6 to 12 months. Adolescents and their parents are asked to take part in study assessments every 3 months during treatment and 3 months after the end of treatment (for an overview of study flow see Fig. 1). The design is adaptive in that both, intervention and scientific assessments can be changed in the course of the study, if, e.g., drop-out rates and reasons indicate necessary adjustments for successful study continuation. Originally, this feasibility trial was designed as a single-blinded RCT to test the effectiveness of MBT-CD compared to treatment as usual (TAU) delivered as an outpatient treatment at the Clinic of Child and Adolescent Psychiatry at the University-Hospital Heidelberg. This comparison group was chosen as it is in line with the routine health care treatment for adolescents with CD [30]. Due to recruitment problems identified in yearly interim recruitment analyses, the design was changed into a non-randomized single group feasibility and pilot trial. This decision was made in consensus with the study funders (Dietmar-Hopp-foundation and Heidehof foundation) upon fulfilling the study termination criterion of recruiting less than 20% of the a priori estimated overall sample size of 102 adolescents within 1 year (to detect a medium effect and considering 25% drop-out).

### Primary outcomes

The feasibility of a prospective RCT on MBT-CD will be estimated based on the acceptability of the intervention and scientific assessments by the participating adolescents as well as the estimated organizational resources needed.

Acceptability of the intervention will be evaluated based on quantitative and qualitative data:

1. Recruitment rates, and consent rates
2. Completion rates
3. Drop-out rates and reasons
4. Treatment duration in months and number of sessions
5. Oral evaluation of the intervention by the patients assessed via standardized questions

Acceptability of the scientific assessments will be evaluated based on quantitative and qualitative data:

1. Adherence rates, missing data
2. Preference of online or paper-pencil assessments
3. Oral evaluation of the assessments by the patients

Resources needed by the organization will be determined regarding:

1. Scientific personnel
2. Recruitment networks, clinical cooperations
3. MBT trainings and supervision

### Secondary outcomes

#### CD symptom severity

1. Fulfillment of CD criteria assessed with the Mini-International Neuropsychiatric Interview for Children and Adolescents (M.I.N.I. KID) [31] and the Structured Clinical Interview for DSM-IV Axis II Disorders (SCID-II) [32]
2. Levels of aggression assessed with the Reactive–Proactive-Aggression Questionnaire (RPQ) [33]
3. Antisocial behavior measured with the Subtypes of Antisocial Behavior Questionnaire (STAB) [34]

#### Mentalizing

1. Mentalizing assessed with the Reflective Functioning Questionnaire (RFQ) [35]
2. The Brief Reflective Functioning Interview (BRFI) [36]
3. The Movie for the Assessment of Social Cognition (MASC) [37]

### Additional assessments

#### Sociodemographic data and childhood experiences

1. Age
2. Gender
3. Attended type of school
4. Childhood and adolescent experiences of neglect and abuse in the family context will be measured with the Childhood Experience of Care and Abuse Questionnaire (CECA-Q) [38]

#### Global and personality functioning

1. Global functioning assessed with the Global Assessment of Functioning Scale (GAF) [39], the Clinical Global Impression – Severity Index (CGI-SI) [40], and the General psychological symptom severity is measured with the Symptom-Checklist-90-Revised (SCL-90-R) [41]
2. Personality functioning assessed with the Levels of Personality Functioning – Questionnaire for Adolescence (LoPF-Q 12-18) [42]
3. Emotion regulation assessed with the Emotion Regulation Questionnaire (ERQ) [43]
4. Personality Pathology assessed with the Dimensional Assessment of Personality Pathology – Basic Questionnaire (DAPP-BQ) [44]
5. Psychopathy-like traits will be assessed with the Youth Psychopathic Traits Inventory (YPI) [45]

#### Experience of patient-therapist and other relationships

1. Adolescents’ experience of the therapy working alliance assessed with the Working Alliance Inventory-Short Revised (WAI-SR) [45]
2. Attachment anxiety and avoidance assessed with the Experiences in Close Relationships Scale-Revised Child-Version (ECR-RC) [46]
3. Experience of parental behavior assessed with the Zürcher Brief Questionnaire for the Assessment of Parental Behaviors (Zürcher Kurzfragebogen zum Erziehungsverhalten, ZKE) [47]

#### Parental mentalizing, stress, and experience of relationships

1. Mentalizing assessed with the Reflective Functioning Questionnaire (RFQ [35])
2. Parental Stress assessed with the Stress Index for Parents of Adolescents (SIPA) [48]
3. Attachment anxiety and avoidance assessed with the Experiences in Close Relationships Scale-Revised (ECR-R) [49]

### Cost-effectiveness

1. Cost-effectiveness assessed with an adapted version of the European version of the client sociodemographic and service receipt inventory (CSSRI-EU) [50]

### Participants

Adolescents with CD or ODD according to the Diagnostic and Statistical Manual for Mental Disorders (DSM) [1] aged between 11 and 18 years are included in the study. Participants are recruited at the participating centers as well as with leaflets (two separate versions for adolescents and parents), which are distributed through multipliers and institutions (e.g., child and youth welfare services, schools, police stations, probation officers). Prospective participants or caretakers can contact the treatment centers via phone (number provided on the leaflet) to indicate their interest, ask questions, and be screened for eligibility criteria through a standardized checklist assessing rule-breaking and defiant behavior. Participants receive a total of 50€ for taking part in the scientific assessments.

### Eligibility criteria

Adolescents are included if their main diagnosis is CD or ODD. The latter is a precursor or milder form of CD, with a pattern of angry, irritable mood, argumentative/defiant behavior, or vindictiveness [1]. The pattern needs to last over the course of at least 6 months and needs to be exhibited in interaction with at least one individual, who is not a sibling. Moreover, the pattern needs to be associated with distress in social contexts or negative consequences for important areas of functioning [33]. Adolescents with CD or ODD are only included if they are between 11 and 18 years of age and both, adolescents and their parents provide written informed consent (forms based on templates of the European General Data Protection Regulation, GDPR [51];). Adolescents are excluded if they have committed sexual offenses, show acute psychotic symptoms, suffer from early or early-onset schizophrenia, have neurological impairments or intelligence severely below average (IQ < 60) as measured with the Culture Fair Intelligence Test (CFT) [52], are non-German-speaking or have other clinical contraindication for outpatient psychotherapy (e.g., acute suicidality).

Concomitant therapies or interventions including hospital stays are permitted, but will be assessed via the CSSRI-EU [50] and reported with the study results.

### Intervention

MBT-CD is a CD-specific adaptation of MBT [53]. The primary goal of MBT-CD is the recovery of the mentalizing capacity in close relationships and along with this, a reduction of symptoms, especially aggressive and antisocial behavior. The treatment duration is 6 to 12 months and consists of up to 30 weekly individual sessions with the adolescent and up to 10 monthly family sessions. At the beginning of the individual sessions, the adolescent’s mentalizing capacity will be diagnosed (diagnostic phase). Before the 30 individual and 10 family sessions, adolescents and their families attend two psycho-educational workshops. After the end of treatment, 3 booster sessions follow. When indicated, youth welfare services are involved.

The psycho-educational workshop (MBT-CD Introductory Workshop, MBT-CD-I) aims to familiarize the adolescent and their family with the mentalizing concept, educate about CD, the MBT-CD treatment goals, and to strengthen therapy adherence. MBT-CD-I focuses on the topics of mentalizing (i.e., what is mentalizing, failures of mentalizing), emotions (i.e., basic emotions, emotion recognition), attachment and identity, and conflicts and boundaries.

**Fig. 2:**
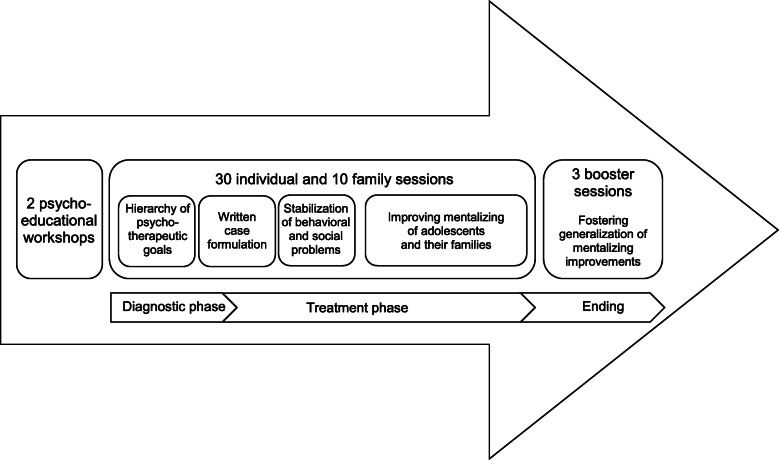
The process of MBT-CD in both settings

After completing MBT-CD-I, adolescents start with their individual sessions. At the beginning of the individual sessions, therapy goals are developed using motivational interviewing and the therapist writes a case formulation about the adolescent’s main mentalizing difficulties, both in collaboration with the adolescent. Moreover, prior to working on improving mentalizing abilities, risk behavior is assessed and a risk emergency plan is developed if necessary. Then, throughout the main treatment phase, MBT-CD focuses on the therapeutic relationship to improve the adolescent’s mentalizing. With the therapist holding the adolescent’s mind in their mind, non-mentalized emotions (rather than cognitions) and their representations can be explored with regard to core problem behavior. MBT-CD focuses on the development of a basic understanding of interpersonal situations, emotions, and failures of mentalizing that trigger antisocial and aggressive behavior. The monthly family sessions (MBT-CD-F) aim to create a mentalizing environment by exploring dysfunctional mentalizing within the family system [54]: With the therapist’s help, family members describe non-mentalizing interactions in detail (noticing and naming), mentalize the interactions (mentalize the moment), formulate potentially dysfunctional family interaction patterns, and consider possible alternatives (generalize and consider change). Reciprocal understanding between therapist and all family members is continuously monitored (checking). This way, MBT-CD-F targets problematic family interactions by practicing mentalizing within relevant interpersonal context and hence enhancing the family’s self-regulatory strategies [54]. At the end of therapy, three booster sessions will follow to stabilize treatment effect (see Fig. 2).

### Treatment adherence

MBT-CD will be delivered by therapists who have undergone psychotherapy training, have the legal right to treat patients under supervision, and who have participated in a 4-day MBT-CD training conducted by the first author (ST). Videos of each session will be obtained. Supervision will be provided in biweekly sessions. During supervision, case material (including therapy videos) is reviewed with regard to the therapist’s understanding of MBT theory and use of MBT interventions. Per therapist, six videos of one of their MBT-CD therapies will be randomly chosen to be rated for therapist adherence and competence with the MBT Adherence and Competence Scale [55].

### Statistical analysis

All analyses will be performed according to the intention to treat principle. Analyses on quantitative data will be conducted using the statistical software SPSS (IBM, Version 25). Sociodemographic data and data of the scientific assessments at baseline (T1, see Table 1) will be used to characterize the sample. Primary outcome variables (recruitment rates, drop-out rates, and missing data of adolescents as well as parents) will be descriptively analyzed. The distribution of treatment duration (in months and number of sessions) will be analyzed to infer the optimal dose of treatment and necessary flexibility in treatment dose and duration. Scientific personnel costs will be estimated based on timely effort (hours needed per month, total number of months). Secondary outcome variables will be investigated using multilevel modeling. Content analysis will be used to investigate qualitative data (drop-out reasons, oral evaluations of MBT-CD, and the scientific assessments). As additional subgroup analysis, binomial logistic regression will be conducted modeled after Jorgensen and colleagues [56], who identified low RF, but not clinical or sociodemographic variables to predict drop-out of MBT group treatment in adolescents with borderline personality disorder. Data entry will be double checked and range checks for data values will be conducted. Pseudonymized data will be anonymized as soon as possible. Anonymized data will be stored in Heidelberg according to the European GDPR [51] and deleted 10 years after study completion.

**Table 1:** Overview of the scientific assessments and time points in both settings

		Measure	T1^a^	T2^b^	T3^c^	T4^d^
Adolescent		WAI-SR		X	X	
		STAB	X	X	X	X
		RPQ	X	X	X	X
		YPI	X			
		CFT-2	X			
		LoPF-Q 12-18	X		X	X
		MASC	X		X	
		RFQ-8	X		X	X
		ECR-RC	X		X	X
		ERQ	X		X	X
		ZKE	X		X	
		BRFI	X		X	
		CECA-Q	X			
		M.I.N.I KID	X		X	
		SCID-II	X		X	
		CGI-SI	X		X	
		SCL-90-R	X		X	
		DAPP-BQ	X			
		GAF	X		X	
		CSSRI-EU	X	X	X	X
Parent		SIPA	X		X	X
		RFQ-8	X		X	X
		WAI-SR		X	X	
		ECR-R	X		X	

*WAI-SR* Working Alliance Inventory-Short Revised, *STAB* Subtypes of Antisocial Behavior Questionnaire, *RPQ* Reactive–Proactive-Aggression Questionnaire, *YPI* Youth Psychopathy Traits Inventory, *CFT-2* Cultural-Fair-Test 2, *LoPF-Q 12-18* Levels of Personality Functioning – Questionnaire for Adolescents, *MASC* Movie for the Assessment of Social Cognition, *RFQ* Reflective Functioning Questionnaire, *ECR-RC* Experiences in Close Relationships Scale-Revised, *ERQ* Emotion Regulation Questionnaire, *ZKE* Zürcher Brief Questionnaire for the Assessment of Parental Behaviors, *BRFI* Brief Reflective Functioning Interview, *CECA-Q* Childhood Experience of Care and Abuse Questionnaire, *M.I.N.I. KID* Mini-International Neuropsychiatric Interview for children and adolescents; *SCID-II* Structured clinical interview for DSM-IV axis II personality disorders, *CGI-SI* Clinical Global Impressions – Severity Index, *SCL-90-R* Symptom-Checklist-90-Revised, *DAPP-BQ* Dimensional Assessment of Personality Pathology – Basic Questionnaire, *GAF* Global assessment of functioning, *CSSRI-EU* European version of the client sociodemographic and service receipt inventory, *SIPA* Stress Index for Parents of Adolescents

^a^Beginning of treatment

^b^3 months after the beginning of treatment

^c^End of treatment

^d^3 months after the end of treatment

## Discussion

This protocol outlines the process of a feasibility and pilot study on MBT-CD in adolescents with CD or ODD. So far, treatments focusing mainly on symptom management remain unsatisfactory in treating patho-mechanisms possibly contributing to CD. Consequently, CD symptoms often persist [12], leading to unfavorable long-term prognoses and, together with insufficient understanding of CD etiology, a pessimism in the treatment of CD [57]. Thus, researchers call for improving CD treatment, e.g., through an integration of knowledge about the etiology and pathological pathways (e.g., [11]). As dysfunctional mentalizing has been linked to the development of CD (cf. [18, 22, 23]), targeting specific mentalizing deficits in this patient group presents a promising approach for a more successful and sustainable treatment of CD and CD (relapse) prevention. However, so far, there are no treatments focusing specifically on mentalizing in adolescents with CD. To fill this gap and aim at a more long-term positive effect on aggressive, norm-violating, and rule-breaking behavior, the authors have developed MBT for CD. Combining individual and family sessions, MBT-CD aims at improving mentalizing in adolescents with CD or ODD and their families within 6 months to 1 year of treatment. In line with the CONSORT statement [58], we aim at investigating the feasibility of an RCT in this patient group in the first step before conducting an RCT investigating its effectiveness in a second step. Several characteristics might render the conduct of an RCT in this patient group especially difficult: e.g., adolescents with CD oftentimes do not express a wish for help or display help-seeking behavior; specific fears may concern losing credibility in their peer group and portraying themselves as “weak.” Moreover, drop-out rates of adolescents with CD are usually high [12], possibly inter alia due to disorganized and avoidant attachment strategies [59]. For this reason, the study design is adaptive, which helps to address CD-specific reservations to the intervention or to the scientific assessments during treatment. Moreover, the consultation of adolescents’ oral evaluations at the end of treatment in addition to the analysis of quantitative data will further help to develop and adapt the treatment in close collaboration with the adolescents. Ultimately, the goal is to provide a treatment, which engages adolescents with CD or ODD and helps fostering the ability to create and maintain healthy relationships and understand triggers of one’s own destructive behavior. As such, MBT-CD is aimed to increase the adolescents’ chances of leading a healthy and satisfying life and along with this, of economically contributing to society.

## Supplementary Information

**Additional file 1..** SPIRIT 2013 Checklist: Recommended items to address in a clinical trial protocol and related documents*

## Data Availability

Pseudonymized data and material will be available in an Open Science Format.

## Abbreviations

ASPD: Antisocial personality disorder
BRFI: Brief Reflective Functioning Interview
CECA-Q: Childhood Experience of Care and Abuse Questionnaire
CFT-2: Cultural-Fair-Test 2
CGI-SI: Clinical Global Impression – Severity Index
DAPP-BQ: Dimensional Assessment of Personality Pathology – Basic Questionnaire
DSM: Diagnostic and Statistical Manual for Mental Disorders
ECR-R: Experiences in Close Relationships Scale-Revised
ECR-RC: Experiences in Close Relationships Scale-Revised for Children and Adolescents
ERQ: Emotion Regulation Questionnaire
GAF: Global assessment of functioning
LoPF-Q 12-18: Levels of Personality Functioning – Questionnaire for Adolescence
MASC: Movie for the Assessment of Social Cognition
MBT: Mentalization-Based Treatment
CD: Conduct disorder
MBT-CD: Mentalization-based treatment for adolescents with conduct disorder
MBT-CDF: Family sessions of mentalization-based treatment for adolescents with conduct disorder
MBT-CDI: Introductory workshop MBT for conduct disorder
M.I.N.I. KID: Mini-International Neuropsychiatric Interview for children and adolescents
RCT: Randomized controlled trial
RFQ: Reflective Functioning Questionnaire
RPQ: Reactive-Proactive-Aggression Questionnaire
SCID-II: Structured clinical interview for DSM-IV axis II personality disorders
SCL-90-R: Symptom-Checklist-90-Revised
SIPA: Stress Index for Parents of Adolescents
STAB: Subtypes of Antisocial Behavior Questionnaire
WAI-SR: Working Alliance Inventory-Short Revised
YPI: Youth Psychopathy Traits Inventory
ZKE: Zürcher Brief Questionnaire for the Assessment of Parental Behaviors

## Declarations

Ethics approval and consent to participate

The Ethics Committee of Heidelberg University (Germany) has approved this study. The study is registered with the number S-534/2016. Written informed consent to participate will be obtained from all participants.
